# Large-Scale Conformational Transitions and Dimerization Are Encoded in the Amino-Acid Sequences of Hsp70 Chaperones

**DOI:** 10.1371/journal.pcbi.1004262

**Published:** 2015-06-05

**Authors:** Duccio Malinverni, Simone Marsili, Alessandro Barducci, Paolo De Los Rios

**Affiliations:** 1 Laboratoire de Biophysique Statistique, École Polytechnique Fédérale de Lausanne, Faculté de Sciences de Base, Lausanne, Switzerland; 2 Structural Computational Biology Group, Spanish National Cancer Research Centre (CNIO), Madrid, Spain; Pierre and Marie Curie University (UPMC), FRANCE

## Abstract

Hsp70s are a class of ubiquitous and highly conserved molecular chaperones playing a central role in the regulation of proteostasis in the cell. Hsp70s assist a myriad of cellular processes by binding unfolded or misfolded substrates during a complex biochemical cycle involving large-scale structural rearrangements. Here we show that an analysis of coevolution at the residue level fully captures the characteristic large-scale conformational transitions of this protein family, and predicts an evolutionary conserved–and thus functional–homo-dimeric arrangement. Furthermore, we highlight that the features encoding the Hsp70 dimer are more conserved in bacterial than in eukaryotic sequences, suggesting that the known Hsp70/Hsp110 hetero-dimer is a eukaryotic specialization built on a pre-existing template.

## Author Summary

Molecular chaperones are a class of proteins that are crucial for the correct functioning of cells. They play central housekeeping roles in the normal cell cycle, and are major actors of the protection system of the cell against cell stress conditions. In this study, we apply statistical inference methods to analyse the structure and function of the Hsp70 molecular chaperone, one of the main members of chaperones. We use the correlated amino acid coevolutions in protein sequences to identify directly interacting amino acids. Our results show that coevolutions capture an appreciable fraction of native contacts throughout the protein. Furthermore, amino acid coevolution predicts previously hypothesized functional dimer interactions between Hsp70s, thus giving a theoretical contribution to this debate.

## Introduction

Molecular chaperones are a broad class of proteins that protect cells against the potentially deleterious effects of denatured and unfolded proteins. They have been shown to play an essential role in multiple proteostasis pathways [1,2]. The 70-kDa heat shock proteins (Hsp70s) are highly conserved and ubiquitous chaperones present in virtually all organisms [3–5]. Besides the canonical roles of chaperones under stressful conditions, Hsp70s have been identified playing several housekeeping roles in the cell under normal conditions such as assisted folding [6,7], oligomeric complex assembly [8], cell cycle regulation [9], import of unfolded polypeptides in the mitochondria and endoplasmic reticulum [10,11], as well as ubiquitin-mediated protein degradation [12,13] and prion propagation [14].

These tasks are all supported by the ability of Hsp70s to bind substrate proteins through an ATP-consuming, non-equilibrium biochemical cycle [15] that involves several conformational transitions at different scales due to nucleotide and substrate binding. The Hsp70 cycle is further regulated by the cooperative action of co-chaperones: J-domain proteins (JDPs) strongly stimulate ATP-hydrolysis [16], whereas nucleotide exchange factors (NEFs) catalyse the release of ADP [17,18].

**Fig 1:**
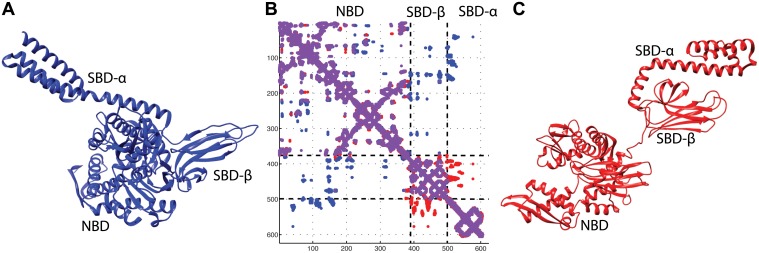
ATP/ADP states of DnaK and the associated contact map. Dashed lines in the map represent the limits of the domains. **A)** Crystal structure of DnaK in ATP state (PDB ID 4jne). **B)** Contact map of the union of both structures. In purple are contacts present in both structures, in red contacts only in ADP, in blue contacts only in ATP. All contacts refer to a threshold of 8.5 Å. **C)** Crystal structure of DnaK in the ADP state (PDB ID 2kho).

Hsp70s are composed of two domains, connected by a flexible linker (Fig 1). The N-terminal ATPase nucleotide-binding domain (NBD), is composed of four lobes, and hosts the active site where ATP and ADP molecules bind. The C-terminal substrate-binding domain (SBD) is subdivided into a β-sandwich subdomain and an α-helical lid; it binds exposed hydrophobic stretches of target substrates in non-native conformations [19–22]. When Hsp70s are bound to ATP, the α-lid and the β-sandwich of the SBD dock onto opposite lobes of the NBD. Upon ATP hydrolysis, the NBD undergoes an intra-domain allosteric transformation resulting into a slight rotation of the lobes with respect to each other [23–25]. Concomitantly, a larger-scale inter-domain allosteric change takes place, whereby the two SBD subdomains undock from the NBD and bind to each other, clamping any substrate that was bound to the β-sandwich.

Beyond these functional conformational rearrangements, oligomerization has been reported for several members of the Hsp70 family [26–29]. Recently, Hsp70 oligomers have been observed by means of electron microscopy [30] and mass spectroscopy [31]. Notably, the results of the latter study indicated that the intermolecular interaction between the linker and the SBD might be responsible for the assembly. Unfortunately neither the functional relevance of these oligomeric states nor the structural details of the quaternary arrangements have yet been clarified.

In contrast, the interaction of Hsp70s in eukaryotes with members of the related Hsp110 family has been well characterized functionally as well as structurally. Hsp110s have been shown to act as a NEFs in the Hsp70 cycle [32,33] and later studies have indicated that in the Hsp70/110 dimer, the two proteins act both as bona-fide chaperones and as mutual NEFs [34]. Indeed, when Hsp70s are bound to ADP, the binding of Hsp110 induces a slight opening of the lobes forming the NBD of Hsp70, thus leading to a facilitated release of the nucleotide [8]. The crystal structure of this complex has been determined [8,35], revealing that the two chaperones associate through both NBD/NBD and NBD/SBD contacts. Intriguingly, similar inter-molecular arrangements have been observed in crystals of DnaK, an *E*. *coli* bacterial Hsp70 [21,22] although their functional relevance has not been determined.

The structure and function of proteins is encoded in their amino-acid sequence, which is constantly under the combined evolutionary action of random mutations and selection. Consequently, it has to be expected that a careful analysis of the Hsp70 sequences across the whole family should reveal the presence of residue pairs that coevolve, *i*.*e*. exhibit correlated mutations, because they are close to each other in the three-dimensional structure and must therefore coordinate their physical properties [36–41]. Based on these premises, the recent Direct Coupling Analysis (DCA) [42–45] has emerged as the most effective algorithm to exploit residue coevolution for the prediction of structural contacts [46,47]. This technique not only can reliably predict the protein native structure [45,48–52], but it can also predict the presence of multiple conformers of the same proteins [44,48,53], homo-multimerization [44,48] and protein-protein interactions [54,55]. DCA, together with similar techniques, has been recently applied to the Hsp70 family by General et al. [56], to study the key residues involved in the allosteric signal propagation in Hsp70 chaperones.

Here we take advantage of the ubiquitous nature of Hsp70s and of the rapid growth of available sequenced proteomes to extensively characterize the function-determining structural features at multiple scales by means of DCA. The structures of the NBD and of the SBD clearly emerge from our analysis, as well as their intra- and inter-domain rearrangements during the chaperone functional cycle. Even more strikingly, DCA predictions show that inter-molecular contacts consistent with crystallographic structures are significantly conserved, thus pointing to their functional relevance.

## Results

To investigate residue coevolution in the Hsp70 family, we built a multiple sequence alignment (MSA) containing 3708 sequences, defining 624 residue positions (see Material and Methods). Our MSA covers almost equally bacterial and eukaryotic sequences (1562 Eukaryotes, 1982 Bacteria). DCA was performed on the MSA using the Pseudo-Likelihood method ([52,57], see Material and Methods) and predicted contacts were ranked according to their DCA scores, which denote coevolution strength between pairs of residues. After discarding contacts at sequences separation less than five, mostly related only to local secondary structures, we retained the 624 DCA contacts with the highest scores (corresponding to 0.325% of the total 191890 possible contacts).

DCA predictions can be compared with the structural information available for *E*. *coli* DnaK, for which high-resolution structures are available for both ATP- (PDB ID 4jne [22] and 4b9q [21]) and ADP-bound states (PDB ID 2kho [20]). Regarding the former state, we use the higher resolution structure (PDB ID 4jne, see S1 Fig for analogous comparison with 4b9q and S1 Text for comments on the supplementary material). The RMSD between the two structures is ~2Å, computed on 597 CA atom pairs, see S8 Fig). Comparisons with partial structures are reported in S3 Fig. We identified 8054 inter-residue native structural contacts (See Material and Methods) in the ATP-bound structure and 7758 in the ADP-bound structure. A close scrutiny of the ATP- and ADP-bound native contact maps of DnaK emphasizes differences between the two nucleotide-bound conformers, which appear as sets of intra- and inter-domain contacts (Fig 1B). In particular, the contacts associated with the ATP-bound structure docking of the lid on the NBD are mutually exclusive with those characterizing the clamping of the α-lid subdomain on the β-sandwich of the SBD in the ADP-bound structure.

**Fig 2:**
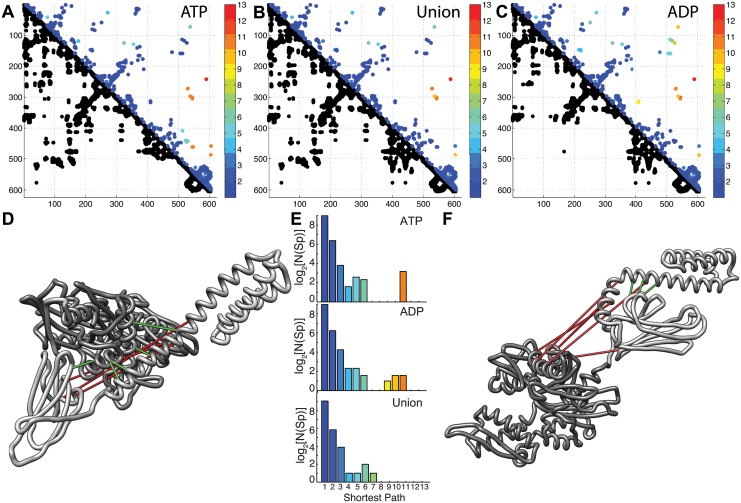
DCA predictions of DnaK allostery. In the contact maps (A-C), the lower triangular parts are the structure contacts at threshold 8.5 Å, the upper parts contain the DCA predictions, coloured by shortest paths. In D and F are shown 8 strongly allosteric contacts. In green are contacts that are true in the conformation, in red contacts that are false in the conformation. The correct contacts in ATP are false in ADP, and vice-versa: The three false positives in the ATP state (red lines) are true positives in the ADP state (green lines). Conversely, the 5 true positives in the ATP state are false positives in the ADP state. **A)** Contact map of DnaK ATP (PDB ID 4jne). **B)** Contact map of the union ATP/ADP. The shortest paths are taken as the minimum between the corresponding shortest paths in the two states. **C)** Contact map of DnaK ADP (PDB ID 2kho). **D)** Set of 8 strong allosteric contacts in the ATP state. 5 correct contacts (green lines), 3 false contacts (red lines) **E)** Histograms of shortest paths of the predicted DCA contacts. Each histogram refers to the corresponding contact map. Counts are reported in log-scale. Bins are coloured corresponding to the colour scheme of the contact maps. **F)** Set of 8 strong allosteric contacts in the ATP state. 3 correct contacts (green lines), 5 false contacts (red lines).

When comparing DCA predictions with the contact maps of the two nucleotide-bound states, we observe a high number of true positives (TPs), *i*.*e*. correctly predicted contacts (see S2 Fig for TP rates). Indeed, 502 out of 624 predicted contacts correspond to native structural contacts in the ATP-bound state, and 504 in the ADP-bound state (Fig 2), resulting into a TP rate of about 80%. Importantly, all the characteristic structural elements shared by both structures, such as the triple-bundle forming the α-lid subdomain, the β-sandwich and the four-lobe structure of the NBD, are well predicted by DCA. To provide a more refined appraisal of the DCA results, each predicted contact is associated with the length of the shortest path (SP) between the corresponding residues, computed over the contact map of the crystallographic structure (see Material and Methods). In this context, the SP provides a topological measure of the distance between two residues that further characterizes our prediction. As shown by Burger and van Nimwegen [58] in the context of mutual information coevolutionary networks, the shortest paths efficiently capture the mediation of coevolutions along chains of residues. Indeed, we observe that about half of the predictions not directly compatible with structural contacts have SP = 2, thus involving two residues that have a native contact with the same amino acid. We expect many of these seemingly wrong predictions to be correct, due to the definition of native contacts that depends on an arbitrary threshold (here 8.5Å) and neglects structural fluctuations. Including all the predictions at SP = 2 in the TPs, the TP rate increases to about 93% in the ADP-bound structure and 94% in the ATP-bound structure. We therefore estimate the actual TP rate to be between 80% and 90%.

The comparison of DCA results with the individual contact maps corresponding to the ADP- and ATP-bound states highlights in both cases a small set of significantly incompatible predictions (SP≥6). However since DCA analysis is expected to capture contacts present in all the functionally relevant conformers [48,59], the most appropriate strategy is to compare DCA predictions with a contact map corresponding to the union of those relative to single-states. In this case, the number of TPs grows to 538 (86%) (95% when taking into account contacts with SP = 2) and the number of significantly incompatible predictions (SP≥6) is decreased. This behaviour indicates that DCA predicts those set of contacts that are found exclusively either in the ATP- or in the ADP-bound structures. In the following, we refer to such contacts as allosteric contacts. As seen in Fig 2A–2C, these contacts correspond to the docking of the α-lid on the NBD in the ATP-bound structure and the clamping of the β-basket by the α-lid in the ADP-bound structure. Furthermore, it must be noted that the predicted allosteric contacts appear early in the score ranking (S1 Table), indicating their evolutionary relevance. These results confirm that DCA applied to the Hsp70 family is able to capture the large-scale allosteric transition of this chaperone that involves variations of inter-residue distances larger than 50 Å.

**Fig 3:**
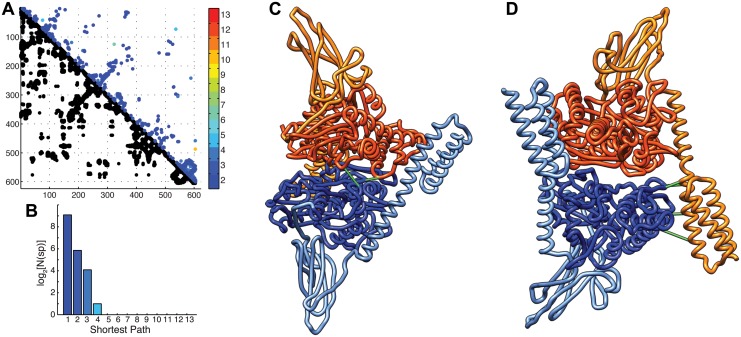
Homo-dimeric complex of *E*. *coli* DnaK. **A)** DCA contact map: The lower triangular part is the structure contacts at threshold 8.5 Å, the upper part contains the DCA predictions, coloured by shortest paths. **B)** Histogram of shortest paths of the predicted DCA contacts. **C-D)** The six dimeric contacts predicted by DCA, illustrated on the ATP state dimer (PDB ID 4jne). Each monomer is coloured by domains, with the Nucleotide Binding domain in darker shade and the Substrate Binding Domain in lighter tones. In C, highlight of the NBD-NBD docking contacts, in D, highlight of the SBD-NBD docking contacts.

A more accurate inspection of the of X-ray structure of DnaK in ATP-bound state (PDB ID 4jne) reveals that the small subset of coevolving pairs of residues not compatible with the monomeric structures (SP>9, see Fig 2B) accurately corresponds to the inter-monomeric contacts in the crystallographic arrangement (Fig 3). We identify six predicted inter-monomeric contacts among the top 624 predictions (S2 Table) which can be separated in two groups: One set of four contacts is associated to the docking of the SBD α-lid of one monomer onto lobe II of the NBD of the other monomer (Fig 3D). The second set is composed of two NBD-NBD contacts (Fig 3C). Among these first six predicted DCA contacts, four display clear electrostatic interactions. We can evaluate the probability that the observed allosteric contacts are the result of random errors by calculating the corresponding p-value (see Material and Methods). The resulting value of 1.44x10^-4^ is a clear hint that the dimeric arrangement observed in DnaK crystals is evolutionary conserved in the Hsp70 family, thus suggesting a functional role.

As discussed in the introduction, Hsp110s are eukaryotic remote homologues of Hsp70s that have retained high sequence similarity [60] and are known to form functional hetero-dimers with Hsp70s with a dimerization pattern extremely similar to that observed in DnaK crystals. It could be argued that the dimer-compatible DCA predictions are due to the presence of interacting eukaryotic Hsp70s and Hsp110s in our MSA. Several arguments can be brought forward to discard this possibility. First and foremost, the Pseudo-Likelihood algorithm used here does not consider couplings between residues belonging to two different sequences in the MSA by construction (see Materials and Methods and [52]), thus not predicting Hsp70/110 dimers. Furthermore, to exclude the possibility that the observed dimerization pattern is a consequence of the presence of Hsp110 sequences, we performed a more stringent filtering of our MSA. To this aim we limit our MSA only to sequences explicitly tagged in Uniprot by the canonical Hsp70 gene names *hspa1a*, *hspa1b*, *hsp70*, *ssa1* and *DnaK*, resulting in a subset containing 1781 sequences. DCA performed on this reduced set (see S5 Fig) resulted in an overall higher noise level, due to lower statistics. However, all the six originally predicted dimeric contacts were retained in the reduced set and an additional dimer-compatible contact appeared in the top 624 predictions. We can therefore safely conclude that coevolutionary analysis predicts Hsp70 homo-dimerization with a quaternary arrangement similar to that observed in the Hsp70-Hsp110 complex (S4 Fig).

**Fig 4:**
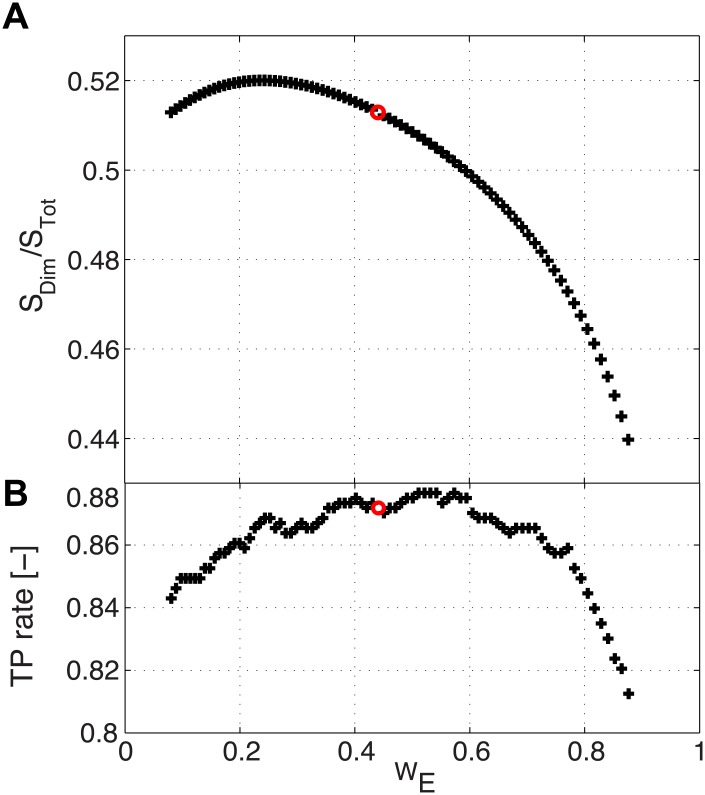
Influence of the relative Bacteria/Eukaryote weights on the dimer contacts. In red are the points corresponding to the unweighted cases. The relative contribution W_E_ is then dictated by the relative abundance of Eukaryotic to total sequences. **A)** Average DCA score for the 6 predicted dimer contacts, normalized by the average over the top 624 predictions. In abscissa is the relative weight of the Eukaryotic sequences in the total alignment. **B)** True positives for the top 624 predictions with respect to the union of ATP-ADP and dimer contacts.

We further investigate if this feature of the Hsp70 family is equally present in the different domains of life. To this aim, we performed DCA artificially varying the relative weights of sequences belonging to eukaryotes and prokaryotes in the MSA. Following this approach, we measured the relative strength of the dimeric contacts as the ratio between their average DCA score and that of the original 624 predicted contacts and we report in Fig 4A this quantity as a function of the weight of eukaryotic sequences in the MSA. The dependence of the TP rate on the same quantity is shown in Fig 4B in order to check if sequence reweighting perturbs the overall quality of the structural predictions. We observe that the relative strength of the dimeric contacts decreases as the eukaryotic weight increases, thus suggesting that Hsp70 homo-dimerization has bacterial origin. This behaviour is observed in a range of relative weights (W_E_ in 0.3–0.7) where the overall quality of prediction is globally unaffected. Moving away from this region, the relative weights are too unbalanced in either direction resulting into poorer statistics and less reliable predictions. The limiting cases of W_E_ = 0 and W_E_ = 1, corresponding to resp. keeping only bacterial or eukaryotic sequences in the MSA, result into high noise levels (see S6 Fig), as the effective number of sequences is strongly decreased. However, we stress that for W_E_ = 0 (only bacterial sequences) DCA still predicts the six dimeric contacts albeit the higher noise levels, whereas the same are absent in the predictions for W_E_ = 1 (only eukaryotic sequences). All these observations strongly suggest that the predicted homo-dimerization of Hsp70s emerges mainly from bacterial sequences whereas this feature is absent or significantly less conserved in eukaryotes.

## Discussion

The function of Hsp70s depends on multiple conformational changes. The structure of the NBD varies with the nature of the bound nucleotide, and the conformational changes induced by nucleotide binding or hydrolysis are propagated to the SBD, thus modulating the Hsp70 interactions with the substrate during the chaperones biochemical cycle. The functional necessity of this orchestrated gymnastic has left a profound footprint in the evolutionary history of these chaperones. It is thus not surprising that sequence analysis methods based on coevolution can effectively provide structural information on all the functional conformers, thanks to the taxonomic breadth of the available Hsp70 sequences.

Indeed, we have found here that out of the first 624 contacts predicted by the Pseudo-Likelihood based method, 75% are compatible with the structures of ATP- and ADP-bound Hsp70s and another 11% can be explained by the intra- and inter-domain allosteric transformations. It is noteworthy that even though DCA has already been used to detect multiple conformers in proteins [44,48,53,56], the analysis of the Hsp70 family presented here resulted into an unprecedented characterization of a large-scale conformational transition due to the identification of an appreciable fraction of relevant allosteric contacts.

Our DCA predictions show thus a remarkable matching (86%) with the contacts between residues in the experimental structures of functional Hsp70 conformers. Furthermore, we introduced here a topological measure inspired from graph theory to further characterize the quality of DCA predictions. This measure allows a finer appraisal than the binary true or false classification of contacts based on a hard cut-off. The shortest path analysis highlights that as many as 95% of our predictions may reasonably correspond to real contacts. These results suggest that the remaining apparently wrong predictions may actually correspond to yet uncharacterized structural features. In this respect, we observe that DCA predicts a group of 6–7 contacts that are compatible with the interface between the two Hsp70 molecules in the DnaK crystal. While it has been noted that the two DnaK monomers in the crystal possess an interface reflecting a molecular dimer, the weak propensity for dimerization *in vitro* questioned the functional relevance of the dimer in the chaperone cycle [22]. Our results indicate that in the Hsp70 family this dimerization interface is evolutionary conserved in a statistically significant way, thus strongly suggesting an important role for the homo-dimer in the cellular function of Hsp70s.

The remarkable similarity between the intermolecular arrangement of the Hsp70/70 homo-dimer and that observed in the functional Hsp70/110 hetero-dimer suggests that the latter might represent an eukaryotic functional specialization of a pre-existing Hsp70/70 homo-dimer. This intriguing hypothesis is actually corroborated by our finding that the co-evolutionary conservation of the dimer interfaces is stronger if bacterial sequences are assigned more statistical weight than eukaryotic ones. Indeed, because bacterial genomes are likely under evolutionary pressure to remain short [61,62], we make the hypothesis that bacteria cannot afford having too many specialized versions of the same protein. As a consequence Hsp70 monomers in the homo-dimer may have to play the same role that the specialized Hsp110s perform in the eukaryotic hetero-dimer.

In this work, we based our analysis on the *a priori* knowledge of the existence of multiple conformers, and the availability of their respective structures. Furthermore, we had at hand a crystallographic homo-dimeric arrangement of two Hsp70 monomers. These data allowed us an in-depth analysis of coevolutionary conservation of structural contacts in DnaK and the prediction of the functional relevance of such a quaternary complex of two bacterial Hsp70s. In general, the blind prediction of multiple conformations or multi-meric arrangements of proteins based solely on coevolutionary information remains an important and challenging problem, whose solution would greatly improve the predictive capabilities of DCA and other coevolutionary methods to the study of previously uncharacterized protein families.

DCA has already shown its impressive potential in reproducing known structural information both at the single protein and at the protein-protein interaction level. The rapid growth of the number of available protein sequences, combined with the improvement of inference algorithms, foreshadow a near future when the use of evolutionary information will be fully exploited as a powerful predictive tool. Thanks to its ubiquity and its evolutionary conservation, together with the state-of-the-art Pseudo-Likelihood optimization method, the Hsp70 family offers a glimpse of these opportunities.

## Materials and Methods

### Multiple Sequence Alignments

Starting from the PFAM seed of the Hsp70 family (*PF00012*), we manually curated it by adding sequences and suppressing gapped positions. The added sequences were chosen to cover a wide range of organisms, stemming from different taxonomy (see S3 Table). The seed was then aligned using MAFFT. The aligned seed was used to build a Hidden Markov Model (HMM) of the family, using the HMMER utility hmmerbuild. The multiple sequence alignment (MSA) was built by running a HMMER search on the Uniprot database. We used the union of the Uniprot Tremble (un-annotated sequences) and Swissprot (annotated) databases for the extraction of the Hsp70 MSA. All utilities were run with default parameters. Our Hsp70 family MSA is available online as supplementary material (S1 Dataset).

### Sequence Filtering

We filtered sequences in the MSA based on their gap contents, keeping only sequences with a maximal gap content of 25% in the final alignment. In order to correct for phylogenetic bias, the MSA was filtered by sequence identity using the HHblits hhfilter utility, allowing a maximum of 90% pairwise sequence identity. An alternative consisting of reweighting the sequences based on their mutual identity leads to nearly identical results. As the DCA computation time grows linearly with the number of sequences in the MSA, we chose to filter the sequences by identity rather than reweighting them.

### Direct Coupling Analysis

Direct Coupling Analysis (DCA) was performed using the symmetric version of the pseudo-likelihood method described in [52], which was first introduced in the context of protein contact prediction by Balakrishnan et al. [57]. DCA is based on the use of the maximum entropy principle, constrained to reproduce the observed single- and two-site amino-acid frequencies, leading to a 21-state (corresponding to the 20 natural amino acids and the gap state) Potts model defined by

$$P(S)\;=\;\frac{1}{Z}e^{\sum_{i=1}^{N}h_{i}(s_{i})\;+\;\sum_{i=1,j>i}^{N-1,N}J_{i,j}(s_{i},\;s_{j})}$$

where ***S*** is a sequence in the MSA, *s*_i_ the amino acid at position *i*, *N* the sequence length, and *h*_*i*_ and *J*_*ij*_ the model parameters to be optimized. The parameters *h*_*i*_ and *J*_*ij*_ are efficiently (but approximately) learned through the numerical optimization of the induced Pseudo-Likelihood with respect to the observed sequences in the MSA [52]. The use of the approximate Maximum Pseudo-Likelihood, in contrast to full Maximum-Likelihood method, allows avoiding the computation of the intractable full partition function **Z**. DCA results come under the form of *N*x*N* matrices. Each entry S_ij_ is computed as the Frobenius norm of the local 21x21 coupling matrix *J*_ij_ and represents the intensity of the evolutionary coupling between residues *i* and *j*. An average product correction [63] is finally applied to correct for entropic effects. In our analysis, we retained the N top pairs having the highest coupling scores S_ij_. The list of the top 624 predicted DCA contacts is available as SI (S2 Dataset).

The optimal regularization parameters of the original method by Ekeberg et al. [52] were used in our study (λ_h_ = 0.01, λ_J_ = 0.01). As the identity filtering is performed in the pre-processing of the MSA, the reweighting was disabled, setting the maximal sequence identity to 100%.

### Backmapping on Structures

The top *N* DCA predictions are compared to the binary contact maps of the available crystal structures. Contact maps are built by considering two residues in contact if the smallest distance between their heavy (non-hydrogen) atoms is lower than 8.5 Å. As the MSA sites are defined only where the HMM defines relevant positions, adjacent columns in the MSA are not necessarily adjacent in the real sequences (gaps/insertions/deletions are present). The DCA predictions are thus aligned to the contact maps of the structures, considering only DCA scores where the crystal structure contains residues. This implies that not all residues in the structures have corresponding positions in the DCA predictions. Conversely, not all DCA predictions correspond to residues in all structures. In the case of comparison between multiple conformations of DnaK (ATP/ADP states), we considered DCA predictions only where both structures have defined residues.

### Shortest Path

In order to assess the quality of predicted contacts, we compute the shortest path between the two residues of the contact. The shortest path (SP) is computed considering the binary contact map as an adjacency matrix of an unweighted and undirected network. Each residue corresponds to a node in the graph, and a link connects two nodes if the corresponding residues are in contact in the protein structure. The shortest path between two residues is the smallest number of links in the graph needed to join two nodes. By definition, physical contacts have SP of 1, while higher SPs indicate a higher topological separation between the residues.

The use of the SP analysis helps highlighting the number of intermediary contacts that would be needed to explain an observed DCA prediction. DCA may not be fully capable of disentangling all indirect correlations in the data, and consequently some residual strong co-evolutionary correlations between residues not in contact in the structure might be found in the predictions. The shortest paths of such predictions give a natural measure of the number of contacts in the native structure through which such a mediated coevolution should propagate in order to be observed. For completeness, we report in S7 Fig the same results using Euclidean distances instead of the shortest paths.

### P-value

To quantify the probability of predicted DCA contacts of being random errors, we used the following *p*-value computation. We introduced a null model where DCA contacts are randomly distributed among all possible pairs. The probability of predicting a set of k correct contacts is thus given by the probability of finding k contacts that exist in the structure, when predicting n total contacts, from a total set of N possible pairs, of which K are contacts in the structure. The proposed null model is thus equivalent to the statistical significance test known as Fisher’s exact test. Mathematically, this is modelled by the hypergeometric distribution, given by

$$p(n,k,N,K)\;=\;\frac{\left(\begin{array}{c} K\\ N \end{array}\right)\left(\begin{array}{c} N-K\\ n-k \end{array}\right)}{\left(\begin{array}{c} N\\ n \end{array}\right)}$$

where $\left(\begin{array}{c} N\\ n \end{array}\right)$ denotes the binomial coefficient. The *p*-value is defined as the probability of the null model to predict k or more true contacts. For a set of n chosen candidate contacts, with k predicted contacts, the *p*-value is thus defined as

$$P_{k}\;=\;\sum_{k\prime =k}^{n}p(n,k\prime ,N,K)$$

In the case of the predicted dimeric contacts, we have thus *k* = 6 dimeric predictions among the *K* = 241 dimeric contacts present in the ATP-state homo-dimeric structure (PDB ID 4jne). We make *n* = 624 total predictions, which can potentially take any of the *N* = 624*623/2 values of the possible contacts. We notice that this is a rather conservative null model, as it does not take in account the fact that among the 624 first predictions, more than 80% are actually correct predictions in the monomeric arrangement of DnaK. Taking this fact in account would drastically decrease the number of predictions *n*, and would thus lead to a sensitively smaller p-value.

During the review process, two important additional experimental studies regarding Hsp70 homo-dimerization were published.

In the first one, Boateng et al. [64] have confirmed the presence of a DnaK homo-dimer with an interface similar to the one reported here.

The second work, by Marcion et al. [65], highlights the fundamental role of the C-terminal region for human Hsp70 homo-dimerization.

## Supporting Information

S1 Fig Top 624 DCA predictions on the ATP-bound structure of Kityk et al. In the lower triangular part are the structure contacts at threshold 8.5 Å, the upper part contains the DCA predictions, coloured by shortest paths.
(EPS)

S2 Fig True positive (TP) rates of the DCA predictions. TPs are defined as the ratio between the number of correctly predicted DCA contacts and the total number of DCA predictions. In red are the predictions mapped on the ADP-bound structure, in blue on the ATP-bound structure, in green on the union. The union map is defined as the minimum of each residue pairs distance between the two states.
(EPS)

S3 Fig DCA predictions on partial structures of Hsp70. In the two representative cases, we considered the top N contacts, where N is the number of residues in the structure. In the lower triangular part are the structure contacts at threshold 8.5 Å, the upper part contains the DCA predictions, coloured by shortest paths. The true positive ratios are computed on the 76 partial structures of Hsp70 in the PDB (41 SBD, 35 NBD). **A)** Top 380 predictions of the NBD of Hsp70 (PDB ID 1s3x). **B)** Top 213 predictions of the SBD of Hsp70 (PDB ID 4hyb). **C)** For the structures of the NBD, we considered the top 400 contacts. **D)** For the structures of the SBD the top 150.
(EPS)

S4 Fig Hsp70 family DCA predictions projected on Hsp70-Hsp110 hetero-dimers. In the lower triangular part are the structure contacts at threshold 8.5 Å, the upper part contains the DCA predictions, coloured by shortest paths. A) Top 534 DCA contacts of the yeast SSE1 (Hsp110 homologue)—Bovine Hsc70 dimer (PDB ID 3c7n). B) Top 624 of the yeast SSE1 (Hsp110 homologue)—Human Hsp70 dimer (PDB ID 3d2e).
(EPS)

S5 Fig Top 624 DCA contacts, using only the Hsp70 tagged sequence of the MSA (resulting in 1781 sequences). In the lower triangular part are the structure contacts at threshold 8.5 Å, the upper part contains the DCA predictions, coloured by shortest paths.
(EPS)

S6 Fig Top 624 DCA contacts, using only the bacterial or eukaryotic sequence of the MSA. In the lower triangular part are the structure contacts at threshold 8.5 Å, the upper part contains the DCA predictions, coloured by shortest paths. A) Bacterial MSA (1982 sequences). B) Eukaryotic sequences (1562 sequences).
(EPS)

S7 Fig DCA analysis reported using Euclidean Distances. From top to bottom: ADP-bound state, ATP-bound state, Union of ADP+ATP bound states, Union of ADP+ATP bound states and ATP-state homo-dimeric contacts.
(EPS)

S8 Fig Alignment of the two ATP state PDB structures 4jne and 4b9q. The two views show a 180° rotated version of the structural alignment between the two structures. The RMSD, computed on 597 overlapping CA atoms is of ~2Å.
(TIF)

S1 Table Allosteric DCA predicted contacts among the first top 624 predictions. (DOCX)

S2 Table The six dimeric contacts predicted among the top 624 DCA contacts in the Hsp70 family. (DOCX)

S3 Table Uniprot sequences IDs used to build the initial seed of the Hsp70 family MSA. (DOCX)

S1 Dataset Multiple Sequence Alignment of the Hsp70 family. (FASTA)

S2 Dataset Top 624 predicted DCA contacts, sorted by decreasing coevolutionary strength. (DAT)

S1 Text Supporting information text. (PDF)
