# Supplementary figures and images for: Large-Scale Conformational Transitions and Dimerization Are Encoded in the Amino-Acid Sequences of Hsp70 Chaperones
Source: PLoS Comput Biol. 2015 Jun 5;11(6):e1004262. doi: 10.1371/journal.pcbi.1004262 (PMC4457872; doi:10.1371/journal.pcbi.1004262)

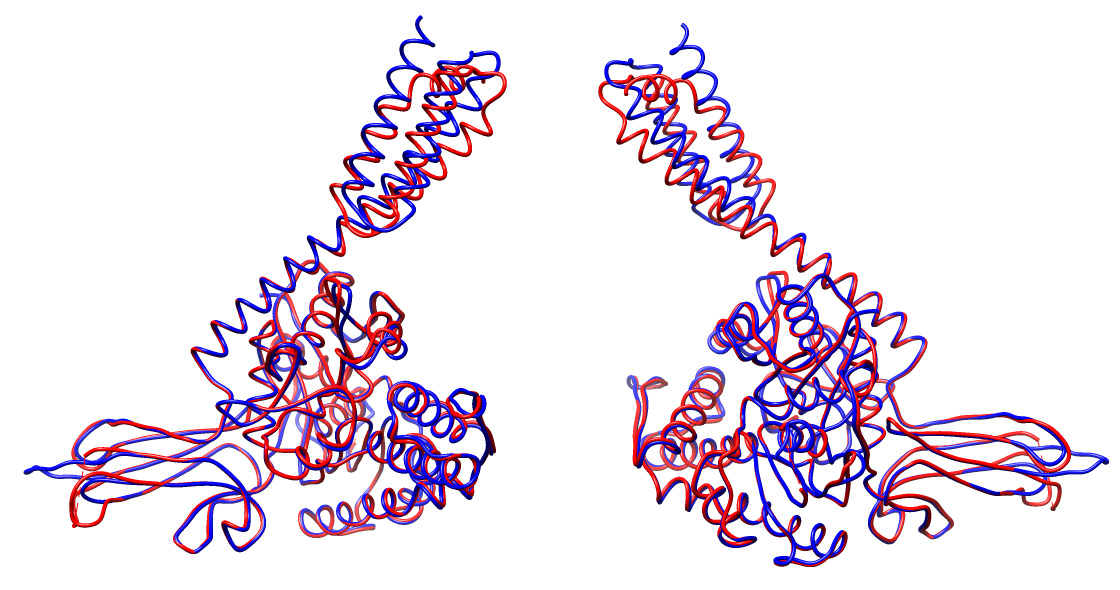

Supplement: S8 Fig — The two views show a 180° rotated version of the structural alignment between the two structures. The RMSD, computed on 597 overlapping CA atoms is of ~2Å. (TIF) [file pcbi.1004262.s008.tif]
