# Supplementary material for: Large-Scale Conformational Transitions and Dimerization Are Encoded in the Amino-Acid Sequences of Hsp70 Chaperones
Source: PLoS Comput Biol. 2015 Jun 5;11(6):e1004262. doi: 10.1371/journal.pcbi.1004262 (PMC4457872; doi:10.1371/journal.pcbi.1004262)
Supplement: S1 Table — (DOCX) [file pcbi.1004262.s009.docx]

**S1 Table.** Allosteric DCA predicted contacts among the first top 624 predictions.

| Rank | Contact | | ATP distance (Å) | ADP distance (Å) |
| --- | --- | --- | --- | --- |
| 52 | Pro256 | Gln63 | 6.51 | 12.69 |
| 54 | Val533 | Arg447 | 54.47 | 6.79 |
| 67 | Glu230 | Ala68 | 4.29 | 10.25 |
| 74 | Lys597 | Ala564 | 8.05 | 9.24 |
| 82 | Asn492 | Asn463 | 8.55 | 2.69 |
| 104 | Lys414 | Val322 | 3.54 | 49.24 |
| 121 | Glu530 | Lys446 | 51.85 | 3.86 |
| 128 | Glu509 | Arg159 | 2.70 | 12.19 |
| 133 | Asn254 | Lys245 | 8.28 | 8.98 |
| 138 | Gln343 | Leu305 | 7.07 | 9.18 |
| 158 | Ile483 | Lys155 | 3.96 | 22.27 |
| 162 | Lys498 | His485 | 11.50 | 5.27 |
| 182 | His226 | Gln150 | 6.48 | 14.83 |
| 200 | Ile190 | Leu181 | 9.05 | 3.43 |
| 201 | Ile338 | Leu181 | 10.39 | 5.39 |
| 205 | Ala480 | Leu382 | 7.36 | 10.67 |
| 206 | Asp490 | Ala465 | 8.61 | 8.45 |
| 210 | Ala370 | Arg362 | 10.77 | 7.64 |
| 226 | Leu382 | Lys166 | 3.82 | 8.69 |
| 227 | Thr225 | Asn147 | 5.24 | 14.37 |
| 229 | Ile601 | Ala553 | 9.47 | 3.73 |
| 235 | Val313 | Glu306 | 6.50 | 9.48 |
| 240 | Met346 | Glu306 | 3.89 | 9.79 |
| 243 | Leu392 | Thr215 | 6.22 | 22.12 |
| 255 | Glu318 | Asp224 | 9.01 | 8.48 |
| 271 | Val516 | Arg159 | 3.94 | 21.80 |
| 280 | Lys548 | Pro466 | 93.39 | 4.64 |
| 285 | Ile499 | Gly455 | 8.68 | 4.43 |
| 290 | Pro134 | Lys125 | 8.35 | 8.69 |
| 292 | Asp561 | Ala553 | 8.36 | 8.85 |
| 294 | Glu530 | Lys125 | 8.00 | 53.13 |
| 295 | Lys414 | Asp326 | 2.65 | 46.42 |
| 311 | Val389 | Leu177 | 4.32 | 17.49 |
| 318 | Ser567 | Ala559 | 8.55 | 7.79 |
| 323 | Thr500 | His485 | 11.41 | 2.78 |
| 338 | Ser595 | Ser545 | 6.56 | 8.70 |
| 339 | Phe529 | Arg447 | 49.08 | 3.42 |
| 343 | Val16 | Gly10 | 8.86 | 8.46 |
| 344 | Lys166 | Arg159 | 8.81 | 8.38 |
| 368 | Val516 | Lys414 | 32.16 | 5.43 |
| 370 | Met599 | Thr546 | 8.73 | 4.67 |
| 382 | Phe529 | Asp450 | 45.25 | 6.98 |
| 393 | Ala480 | Leu390 | 10.23 | 6.33 |
| 404 | Gln513 | Arg159 | 3.76 | 16.05 |
| 409 | Met587 | Leu532 | 7.77 | 8.52 |
| 411 | Asn522 | Glu118 | 3.39 | 46.88 |
| 443 | Ala571 | Thr563 | 8.83 | 8.40 |
| 447 | Thr500 | Asp460 | 5.94 | 14.77 |
| 448 | Gln603 | Lys556 | 9.60 | 7.23 |
| 449 | Lys581 | Gln538 | 8.58 | 7.51 |
| 459 | Met515 | Gly506 | 8.19 | 9.85 |
| 468 | Thr500 | Asn458 | 2.83 | 10.03 |
| 469 | Glu600 | Ile565 | 10.65 | 8.42 |
| 475 | Ser234 | Ile88 | 4.44 | 12.59 |
| 479 | Gln343 | Val309 | 6.80 | 9.73 |
| 484 | Met515 | Gln114 | 3.36 | 37.28 |
| 502 | Asp526 | Arg445 | 42.52 | 3.34 |
| 505 | Ala503 | Ser453 | 4.87 | 12.33 |
| 510 | Val594 | Ala586 | 8.88 | 8.48 |
| 511 | Asn458 | Ser434 | 8.57 | 8.38 |
| 517 | Glu531 | Ala523 | 9.52 | 7.62 |
| 520 | Asp85 | Ile73 | 8.55 | 7.06 |
| 521 | Ala553 | Ser545 | 9.11 | 7.86 |
| 542 | Glu511 | Lys452 | 14.67 | 2.32 |
| 547 | Arg56 | Glu31 | 9.53 | 7.50 |
| 551 | Asp336 | Gly186 | 7.11 | 10.59 |
| 564 | Glu600 | Gln593 | 6.77 | 9.53 |
| 593 | Lys352 | Val337 | 8.53 | 8.48 |
| 613 | Arg527 | Lys446 | 51.38 | 5.06 |
| 620 | Gln589 | Lys581 | 8.86 | 8.02 |
