# Supplementary material for: Large-Scale Conformational Transitions and Dimerization Are Encoded in the Amino-Acid Sequences of Hsp70 Chaperones
Source: PLoS Comput Biol. 2015 Jun 5;11(6):e1004262. doi: 10.1371/journal.pcbi.1004262 (PMC4457872; doi:10.1371/journal.pcbi.1004262)
Supplement: S2 Table — (DOCX) [file pcbi.1004262.s010.docx]

**S2 Table.** The six dimeric contacts predicted among the top 624 DCA contacts in the Hsp70 family.

| Rank | Contact | Distance (Å) |
| --- | --- | --- |
| 185 | Gln277-Gln534 | 5.61 |
| 497 | Glu306-His541 | 4.59 |
| 534 | Asp129-Lys363 | 3.12 |
| 549 | Glu310-Lys548 | 7.06 |
| 590 | Ala30-Ala276 | 6.33 |
| 600 | Gln278-Val533 | 3.32 |
