# Supplementary material for: Large-Scale Conformational Transitions and Dimerization Are Encoded in the Amino-Acid Sequences of Hsp70 Chaperones
Source: PLoS Comput Biol. 2015 Jun 5;11(6):e1004262. doi: 10.1371/journal.pcbi.1004262 (PMC4457872; doi:10.1371/journal.pcbi.1004262)
Supplement: S3 Table — (DOCX) [file pcbi.1004262.s011.docx]

**S3 Table.** Uniprot sequences IDs used to build the initial seed of the Hsp70 family MSA.

| **O73885**  **P08107**  **P0A6Y8**  **P0A6Z1**  **P0C922**  **P0CS91**  **P11021**  **P16394**  **P19378**  **P20030**  **P20442**  **P20583**  **P22010**  **P22358**  **P22774**  **P24067**  **P25840**  **P26791**  **P27541**  **P27542**  **P29215**  **P29845** | **P30722**  **P36604**  **P38646**  **P47773**  **Q01899**  **Q02028**  **Q03685**  **Q05746**  **Q05981**  **Q06248**  **Q1IEI8**  **Q28222**  **Q2S307**  **Q3SEX4**  **Q3YZ26**  **Q5NVM9**  **Q7MNF8**  **Q8H1B3**  **Q8T869**  **Q91291**  **Q9TUG3**  **A1DB02** | **A4RSV4**  **A5AT21**  **A7LH89**  **A7SG65**  **A7TL70**  **A7YVD5**  **A9U4N3**  **B2D2G5**  **B2ZR74**  **B6F134**  **B7U9Z3**  **C4JI41**  **C8ZG23**  **D1FX74**  **D1J774**  **D3AY31**  **D7GL49**  **E1AQZ2**  **E3VXC7**  **F2QWI6**  **F6K6F4**  **F6Z1G9** | **F6ZXI7**  **G1MSW3**  **G2HZX1**  **G3W9P3**  **G9DA09**  **H2B1P0**  **H2B652**  **H2L3G7**  **I1LQR4**  **I1R2A1**  **J3MYR8**  **J7F7M8**  **K0KZP2**  **K1QG22**  **K3Z4C1**  **K4G024**  **K7IQ77**  **M9N2X3**  **O42808**  **Q0KKB3**  **Q17UC1**  **Q22C26** | **Q22CG9**  **Q2PPI9**  **Q3S348**  **Q40151**  **Q5MGA8**  **Q5QHT4**  **Q804B6**  **Q8AYL6**  **Q8UUJ8**  **Q8UV14**  **Q94805**  **Q95V47**  **R9WNY5**  **T1DQ26**  **T1IUX6**  **T2MH32**  **V4AFV6**  **V5FLC8**  **V5RG72**  **W5KA74**  **W7XEU7**  **W8GMB0** |
| --- | --- | --- | --- | --- |
