# Supplementary material for: Large-Scale Conformational Transitions and Dimerization Are Encoded in the Amino-Acid Sequences of Hsp70 Chaperones
Source: PLoS Comput Biol. 2015 Jun 5;11(6):e1004262. doi: 10.1371/journal.pcbi.1004262 (PMC4457872; doi:10.1371/journal.pcbi.1004262)
Supplement: S1 Text — (PDF) [file pcbi.1004262.s014.pdf]

Supplementary Material

1. DCA results on the 4b9q structure

We repeated the DCA analysis on the *E.Coli* DnaK structure in ATP-bound state by Kityk et al. (PDB ID 4b9q [1]) for comparison with the structure of Qi et al. (PDB ID 4jne [2]). As seen in S1 Figure both structures are highly similar and DCA predictions are identical up to some varying contacts. Therefore, we performed the main analysis with the more recent structure from Qi et al.

2. True positive rates for the predictions on the ADP-bound, ATP-bound and Union Structure

In S2 Figure we report the evolution of the ratio of positively predicted DCA contacts (True Positive ratio) with increasing number of predictions, in the ADP- and ATP-bound structures, as well as in the union of both contact maps. We observe that the overall true positive ratio is high for the 624 top contacts used in the main text (>80%). Moreover, when considering the union of both contact maps, we see that the TP rates increase consistently with the predictions of allosteric contacts.

3. List of predicted allosteric contacts in the DCA analysis

S1 table lists the 70 allosteric contacts predicted among the top 624 DCA contacts in the Hsp70 family. Allosteric contacts are defined as pairs of residues that have distances below the threshold (8.5Å) in either one of the ADP- or ATP-bound states, but not in the other. Residue numbering corresponds to *E.Coli* DnaK (Uniprot ID P0A6Y8).

4. DCA analysis on the set of available partials structures of Hsp70s

We repeated the DCA analysis on the set of available partial structures of the Hsp70 family deposited in the PDB. We extracted a total set of 76 structures, composed of 41 structures of the Substrate Binding Domain (SBD) and 35 structures of the Nucleotide Binding Domain (NBD) of Hsp70. In S3 Figure A-B are reported two representative DCA predictions from these respective subsets (S3A: Nucleotide Binding Domain, S3B: Substrate Binding Domain). For each structure, we computed the fraction of correctly predicted contacts, taking the top 400 contacts for the NBD structures and the top 150 for the SBD structures. As seen in S3 Figure C-D, the TP ratios are overall high and consistent with those found for the structures used in the main text.

5. Dimeric interface in the Hsp70-Hsp110 heterodimers

We projected the DCA predictions of the Hsp70 family on contact maps of the known crystalized Hsp70-Hsp110 heterodimers. This highlights the high similarity between the dimerization pattern of Hsp70 homo-dimers and Hsp70-Hsp110 heterodimers, as seen in the dimer contacts in S4 Figure.

6. DCA predictions using a subset of Hsp70 tagged sequences in the MSA

In order to certify that the DCA predictions of the dimeric contacts was not an artefact introduced by the presence of potential Hsp110 sequences in the MSA, we repeated the DCA analysis using a subset of the MSA containing sequences with explicit Hsp70 gene names in the Uniprot database, namely *hspa1a, hspa1b, hsp70, ssa1* and *DnaK*. This resulted in a reduced MSA containing 1781 sequences. Having reduced by a factor ~2 the number of sequences, the method has less data to build representative amino acid occurrences and co-occurrences statistics to fit the Pseudo-Likelihood parameters. The overall noise level of the predictions therefore increases, as seen in S5 Figure. The dimeric contacts are however still present among the top 624 predicted DCA contacts.

7. DCA predictions using only Bacterial or Eukaryote tagged sequences in the MSA

When reweighting the sequences based on their belonging to bacteria or eukaryotes, the two limiting cases corresponding to $W_E = 0$ and 1 correspond respectively to making DCA predictions using only bacterial resp. eukaryotic sequences. In the first case (bacteria only) the resulting MSA contains 1982 sequences, in the second case (eukaryotes only) the MSA contains 1562 sequences. The consequences of this drastic reduction in input data is seen on the noise levels of the resulting predicted contacts (Fig. S6). Nevertheless, we observe that the dimeric signal is still present in the bacterial DCA (S6 Figure A), while it disappears in the eukaryotic DCA (S6 Figure B).

8. List of predicted Dimer contacts in *E.Coli* DnaK

We report hereafter (S2 Table) the six predicted dimeric contacts in the Hsp70 homo-dimer. Residue numbering corresponds to *E.Coli* DnaK (Uniprot ID P0A6Y8).

9. List of Uniprot Ids of the sequences used in the seed for generating the MSA

We report hereafter (S3 Table) the Uniprot Ids of the sequences in the seed used to extract Hsp70 sequences from the Uniprot/Swissprot database.

10. DCA analysis reported using Euclidean distances

In S7 Figure, we report the results presented in the main text using Euclidean distances in place of the shortest paths. From top to bottom, the maps and histograms correspond to : ADP-bound DnaK, ATP-bound Dnak, Union of ADP+ATP and Union of ADP + ATP + ATP dimeric contacts.

11. Comparison of the two crystal structures of the ATP-bound state DnaK

In S8 Figure, we present the structural alignment of PDB ID 4jne [2] and PDB ID 4b9q [1], resulting in a RMSD of ~2Å.

12. DCA datasets

Supplementary material S1 Dataset contains the multiple sequence alignment used to perform the coevolutionary analysis of the Hsp70 family. S2 Dataset contains the list of the 624 highest ranked DCA contacts considered in this article.

References

1. Kityk R, Kopp J, Sinning I, Mayer MP (2012) Structure and dynamics of the ATP-bound open conformation of Hsp70 chaperones. Mol Cell 48: 863–874.

2. Qi R, Sarbeng EB, Liu Q, Le KQ, Xu X, et al. (2013) Allosteric opening of the polypeptide-binding site when an Hsp70 binds ATP. Nat Struct Mol Biol 20: 900–907.
